# Probiotics for ulcerative colitis: mechanisms, therapeutic advances, and emerging strategies

**DOI:** 10.3389/fmicb.2026.1787284

**Published:** 2026-03-17

**Authors:** Chao Niu, Jing Wang, Xinping Lu, Yongduo Yu

**Affiliations:** 1 The Second Clinical College, Liaoning University of Traditional Chinese Medicine, Shenyang, China; 2 Changzhi Hospital of Traditional Chinese Medicine, Changzhi, China; 3 The Second Affiliated Hospital of Liaoning University of Traditional Chinese Medicine, Shenyang, China

**Keywords:** gut microbiota, immunomodulation, probiotics, targeted delivery, ulcerative colitis

## Abstract

Ulcerative colitis (UC) is a chronic, relapsing inflammatory bowel disease with a rising global incidence. Existing therapies are often limited by suboptimal efficacy and frequent relapse. Gut microbiota dysbiosis is central to UC pathogenesis, providing a rationale for probiotic-based, microbiota-targeted interventions. This review synthesizes evidence that probiotics mitigate UC through multiple synergistic mechanisms: competitive exclusion to rebalance the microbiota, upregulation of tight junction proteins to restore the intestinal barrier, and bidirectional immunomodulation to restrain excessive inflammation. These mechanisms are supported by extensive preclinical and clinical data. Research is increasingly moving beyond conventional live formulations toward defined postbiotics, probiotic–traditional Chinese medicine combinations, targeted delivery systems engineered with smart materials, and fecal microbiota transplantation. These strategies seek to overcome limitations such as low bacterial viability and poor targeting, thereby improving therapeutic precision and efficacy. Collectively, probiotics and their derivative approaches offer promising adjunct or alternative options for the clinical management of UC via multitarget modulation of the intestinal microenvironment.

## 1 Introduction

Ulcerative colitis (UC) is a chronic, relapsing inflammatory disorder of the colon and rectum, characterized by abdominal pain, diarrhea, hematochezia, and tenesmus. The disease often follows a protracted course, substantially impairing quality of life and imposing a significant healthcare burden (Segal et al., 2021; Wangchuk et al., 2024). Its epidemiology shows marked geographic variation: incidence is highest in North America and Western Europe (9–20 cases per 100,000 person-years) but is rising rapidly in regions historically considered low risk, including parts of Asia and South America (Hracs et al., 2025; Kaplan and Windsor, 2021).

Pharmacologic management of UC aims to control inflammation and induce and maintain remission. Core agents include 5-aminosalicylic acid (5-ASA) compounds, corticosteroids, immunosuppressants, and biologics (Sebastian et al., 2024; Wan et al., 2025). However, efficacy is variable, adverse effects can be substantial, and relapse rates remain high (Yiu et al., 2024). These limitations underscore the need for novel therapeutics that target more upstream pathways in UC pathogenesis.

In recent years, gut microbiome research has provided new insights into the pathogenesis and treatment of UC. The gut microbiota, a critical interface mediating host-environment interactions, is essential for maintaining intestinal homeostasis. Dysbiosis—an imbalance in this ecosystem—has been implicated as a key driver of the initiation and persistence of mucosal immune inflammation in UC (Guo et al., 2020). Patients with UC commonly exhibit a depletion of beneficial commensals, including short-chain fatty acid-producing taxa, alongside an expansion of potentially pathogenic species. This disruption compromises epithelial barrier integrity and impairs immune tolerance, thereby triggering and amplifying inflammatory cascades (Zhang H. et al., 2025; Zou et al., 2021). Consequently, probiotic therapy—supplementation with live beneficial microorganisms to restore microbial balance—has emerged as a promising disease-modifying approach. Probiotics act through multiple mechanisms, including reshaping microbial communities, strengthening the intestinal barrier, and modulating host immune responses (Beikmohammadi et al., 2025; Rana and Smriti, 2025). They are generally well tolerated with favorable safety profiles, supporting high patient acceptability (Almutawif et al., 2025).

Despite this promise, the clinical efficacy of probiotics varies substantially across strains and individuals. A systematic appraisal of mechanisms, applications, and strategies to overcome current limitations is therefore essential to advance clinical translation. This review synthesizes high-quality evidence from the past 5 years, detailing the principal mechanisms by which probiotics modulate the gut microbiota to ameliorate UC and summarizing advances from animal and clinical studies. It also provides a focused discussion of emerging approaches—including postbiotics, probiotic–traditional Chinese medicine (TCM) combinations, targeted delivery systems, and fecal microbiota transplantation (FMT)—providing a conceptual framework and new directions for developing effective, durable next-generation microecological therapies.

## 2 Disease concept and pathogenesis of UC

UC is a chronic, relapsing–remitting inflammatory disorder of the colonic and rectal mucosa. Clinically, it presents with recurrent diarrhea, bloody, mucopurulent stools, abdominal pain, and tenesmus, and may be accompanied by extraintestinal manifestations such as arthritis and cutaneous lesions (Magro et al., 2017). The disease alternates between active and remission phases. Long-standing active disease increases the risk of colorectal cancer, and severe cases can lead to life-threatening complications, including toxic megacolon and intestinal perforation (Dao et al., 2024; Rubin et al., 2025).

**Figure 1:**
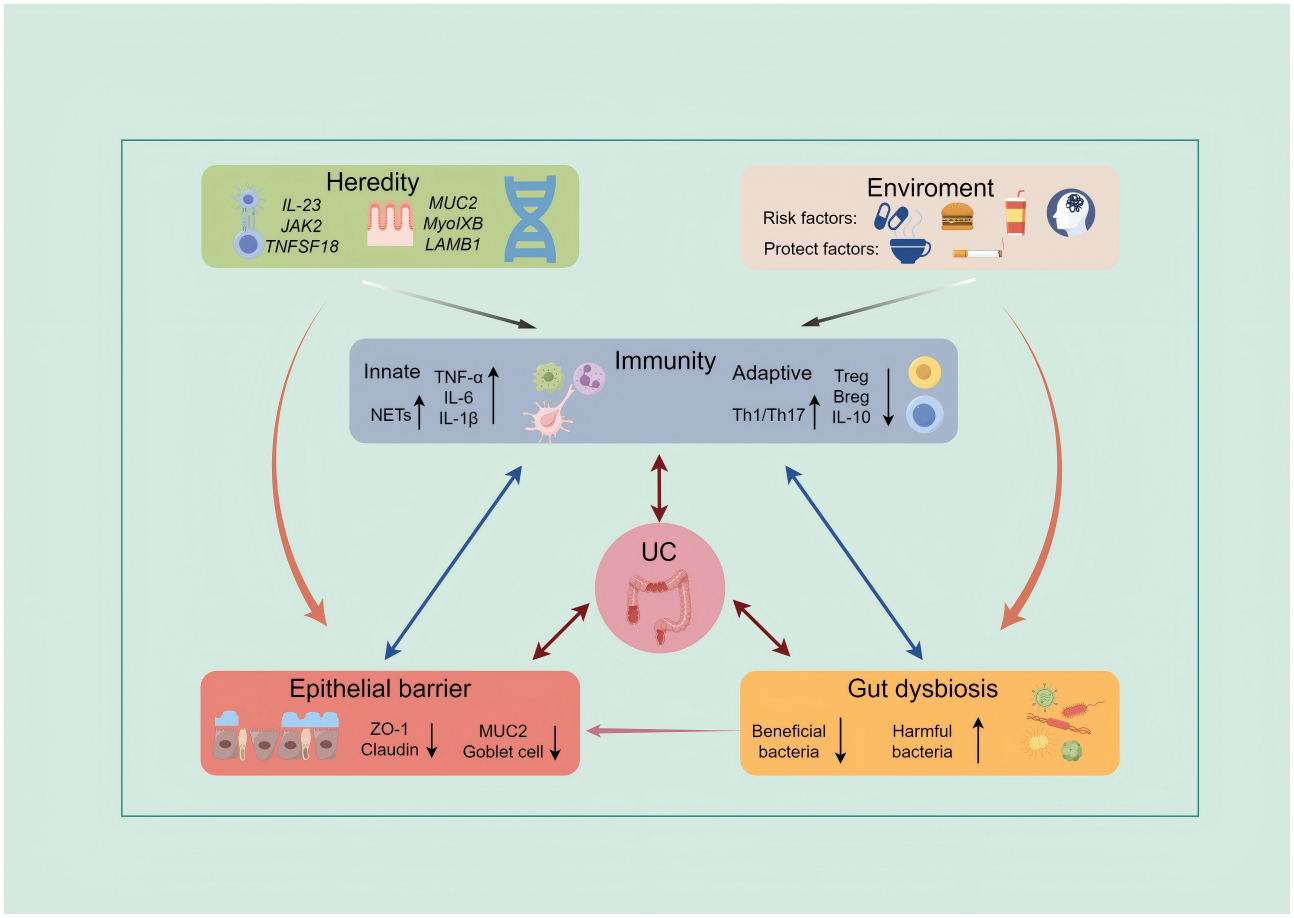
Schematic diagram of the pathogenesis of UC. The development of UC involves multiple interacting factors, including genetic susceptibility, environmental triggers, immune dysregulation (both innate and adaptive), impaired epithelial barrier function, and gut microbiota dysbiosis. These elements collectively drive chronic intestinal inflammation. By Figdraw.

UC pathogenesis is a multistep interplay among genetic susceptibility, environmental exposures, microbial dysbiosis, epithelial barrier dysfunction, and mucosal immune dysregulation (Agrawal and Gupta, 2025; Caba et al., 2025). Genetic background confers susceptibility; environmental factors (e.g., diet, medications, infections) disrupt the gut microbiota; and the resulting dysbiosis, together with barrier defects, increases immune exposure to luminal antigens. This provokes a persistent, dysregulated immune-inflammatory response against commensal microbes and/or self-antigens, ultimately leading to tissue injury and clinical symptoms (Figure 1).

### 2.1 Genetic factors and ethnic variations

Genetic predisposition is a major contributor to UC. Genome-wide association studies and sequencing have identified more than 260 susceptibility loci for inflammatory bowel disease (IBD), enriched for genes involved in innate and adaptive immunity and immune signaling (e.g., *IL23R, JAK2, TNFSF18*; Porter et al., 2020). Some loci are shared between UC and the other major form of IBD, Crohn's disease (CD), whereas others show disease-specific associations. The strongest associations map to the human leukocyte antigen (HLA) region, particularly HLA class II (e.g., HLA-DRB1), with at least 16 HLA alleles linked to UC (Naito and Okada, 2022; Zhu et al., 2020). For example, carriers of the HLA-DRB1^*^01:03 allele (approximately 3% of patients) have a greater than 40% risk of requiring major intestinal surgery within 3 years of diagnosis (Vestergaard et al., 2024). Variants in epithelial barrier genes (e.g., *MUC2, LAMB1, MyoIXB*) can also increase intestinal permeability and thereby heighten UC susceptibility (Li et al., 2016).

Genetic susceptibility to UC varies substantially across ethnic and geographic groups. In East Asian cohorts, variants in *ADAP1* and *GIT2* are significantly associated with UC risk (Liu Z. et al., 2023). By contrast, among Ashkenazi Jews, variants in *IRF5, TLR4*, and *VDR* are linked to an increased risk of UC (Wu et al., 2023b). These findings highlight population-specific genetic architectures and underscore the need to incorporate ancestry into risk prediction and precision-medicine strategies.

### 2.2 Environmental factors

Multiple environmental exposures contribute to the onset and progression of UC by altering gut microbiota composition, compromising epithelial barrier integrity, and modulating mucosal immunity. Medication exposures are among the best-established risks: repeated antibiotic use disrupts microbial homeostasis, and non-steroidal anti-inflammatory drugs (NSAIDs) can damage the intestinal mucosa (Preda et al., 2019). Diet is another key, modifiable determinant. Long-term adherence to a Western dietary pattern—high in fat and refined sugar and low in fiber—is associated with an increased risk of UC, potentially via altered bile acid metabolism and increased intestinal permeability (Schreiner et al., 2019). Notably, the incidence of cholangiocarcinoma is substantially higher among patients with UC than in the general population (OR = 7.67; 95% CI, 6.96–8.46), suggesting that unhealthy dietary patterns may further increase the likelihood of progression from UC to cholangiocarcinoma through pathways such as chronic inflammation (Dominguez et al., 2024). In contrast, polyphenol-rich diets (e.g., regular tea consumption) appear to confer protective effects (Nie and Zhao, 2017). Chronic alcohol intake directly compromises intestinal barrier function by inducing oxidative stress, positioning ethanol as a potential upstream driver in UC pathogenesis (Chung et al., 2025). The relationship with smoking is complex. Epidemiological studies suggest that nicotine and other constituents may exert anti-inflammatory effects and partially reduce risk (Lo Sasso et al., 2020); however, any potential benefit is outweighed by the well-documented harms of smoking, and its long-term net effect on UC remains uncertain (Yan Ang et al., 2024). Psychological factors also exert a profound influence on intestinal health. Chronic psychological stress can influence gut physiology and immunity via the gut-brain axis, creating a bidirectional relationship with disease activity (Frolkis et al., 2019). Finally, prolonged exposure to environmental pollutants, including fine particulate matter (PM2.5), may increase UC risk by promoting systemic inflammation and oxidative stress (Gao W. et al., 2025). Likewise, agricultural inputs such as pesticides and fertilizers, ingested through food or drinking water, may contribute to UC pathogenesis either through intestinal microbial transformation or via direct toxic effects (Fu et al., 2022; Luo et al., 2021; Roediger, 2008).

### 2.3 Gut microbiota dysbiosis

Dysbiosis of the gut microbiota is a hallmark of UC. Extensive evidence shows that the intestinal microbiota in UC differs markedly from that of healthy individuals, with depletion of beneficial commensals and expansion of potentially harmful taxa (Hansen et al., 2010; He T. et al., 2025; Table 1).

**Table 1:** Changes in the gut microbiota of patients with UC.

**Subjects**	**Population**	**Technique**	**Increased in UC**	**Decreased in UC**	**Reference**
60 UC, 20 HC	China	16S rRNA (V3–V4)	Proteobacteria, *Streptococcus, Faecalicoccus*	*Lactobacillus, Butyricicoccus, Lachnospira, Phascolarctobacterium*	(Zhu et al. 2022)
62 UC, 31 HC	China	16S rRNA sequencing	Proteobacteria, Gammaproteobacteria, Enterobacteriaceae	*Bifidobacterium*, Clostridiales	(Chen et al. 2021)
37 UC, 13 HC	Hungary	16S rRNA (V4)	Pasteurellaceae, Enterobacteriaceae, Enterococcaceae	*Bifidobacterium*, Bacteroidaceae, Porphyromonadaceae, Prevotellaceae	(Bálint et al. 2020)
131 UC, 40 HC	South Korea	16S rRNA (V3–V4)	Peptostreptococcacea, Bacilli	Bacteroidetes, Prevotellaceae, Rikenellaceae	(Oh et al. 2024)
10 UC, 10 HC	Poland	16S rRNA (V3–V4)	*Scherichia-Shigella, Peptostreptococcus, Bacillus*	*Akkermansia, Faecalibacterium, Bifidobacterium*	(Zakerska-Banaszak et al. 2021)
421 UC (104 active, 317 remission)	US	ITS2 sequencing	*Candida*	*Agaricus, Rhodotorula*	(Jangi et al. 2024)
40UC, 38HC	US	ITS2 sequencing	*Candida*	*Saccharomyces*	Li W. et al. (2022)

Taxonomic names at the genus or species level are italicized. UC, ulcerative colitis patients; HC, healthy controls.

Across multiple independent cohorts (Table 1), UC is consistently associated with increased relative abundance of Proteobacteria—particularly the *Enterobacteriaceae*—alongside reduced levels of health-associated groups such as *Bifidobacterium*, Lachnospiraceae, and Verrucomicrobia (e.g., *Akkermansia*; Bálint et al., 2020; Chen et al., 2021; Oh et al., 2024; Zakerska-Banaszak et al., 2021; Zhu et al., 2022). Notably, short-chain fatty acid (SCFA)-producing bacteria, especially butyrate producers like *Roseburia* and *Faecalibacterium*, are depleted (Štofilová et al., 2022). Butyrate is the primary energy source for colonocytes and is critical for epithelial barrier integrity and immune tolerance; its deficiency compromises mucosal anti-inflammatory and reparative functions (Zhu et al., 2022).

Fungal dysbiosis (the mycobiome) is increasingly recognized in UC. Both patients and animal models show reduced intestinal fungal diversity and an increased abundance of potentially pathogenic taxa, particularly *Candida albicans* (Jangi et al., 2024; Wang T. et al., 2016). *C. albicans* exacerbates intestinal inflammation by inducing host production of pro-inflammatory mediators, including IL-1β, and its abundance correlates with disease activity. In animal models, blocking IL-1 signaling (e.g., with anti-IL-1R antibodies) ameliorates *Candida*-exacerbated colitis (Li X.V. et al., 2022). Collectively, these findings indicate that specific pathogenic members of the gut microbiota—including bacteria and fungi—actively drive UC pathogenesis through direct and immune-mediated mechanisms.

### 2.4 Impaired epithelial barrier function

Impaired intestinal epithelial barrier function is a central pathogenic feature of UC and evolves over the disease course. Early in disease, epithelial cells may appear morphologically intact, yet increased apoptosis and disruption of tight junctions produce focal increases in permeability. With persistent inflammation, these changes progress to the characteristic mucosal erosions and ulcerations (Bu et al., 2025; Vargas-Robles et al., 2019).

The molecular basis is multifaceted. Structurally, downregulation of key tight junction proteins (e.g., occludin, claudins) together with upregulation of pore-forming proteins (e.g., claudin-2) directly weakens the physical barrier (Górecka et al., 2024; Heinemann and Schuetz, 2019; Hu et al., 2021). At the regulatory level, aberrant activation of canonical pro-inflammatory pathways such as NF-κB disrupts junctional complexes and induces cytokines, including IL-1β, thereby amplifying inflammation and further compromising barrier integrity—a self-perpetuating cycle (Zeng et al., 2024).

Recent work has identified additional regulatory mechanisms. For example, the mechanosensitive ion channel Piezo1 is markedly upregulated in the colonic mucosa of patients with UC (Zhou et al., 2025a), and its excessive activation exacerbates epithelial injury, in part by promoting ferroptosis (He H. et al., 2025; Zhu J. et al., 2024).

### 2.5 Immune dysfunction

In UC, intestinal inflammation is driven by sustained dysregulation of both innate and adaptive immunity, which together propagate and amplify mucosal injury (Kałużna et al., 2022; Yue et al., 2024).

Within the innate compartment, neutrophils, macrophages, and dendritic cells are aberrantly activated (Tang et al., 2025). Via pattern-recognition receptors, these cells sense pathogen- and damage-associated molecular patterns (PAMPs and DAMPs), triggering the production of proinflammatory cytokines (e.g., TNF-α, IL-1β, IL-6) and chemokines that drive the inflammatory cascade (Kałużna et al., 2022). Neutrophils dominate the early infiltrate; although their phagocytic activity and release of neutrophil extracellular traps (NETs) contribute to pathogen clearance, excessive activation and dysregulated NET formation cause direct tissue injury (Dinallo et al., 2019; Long et al., 2024). Macrophages and dendritic cells perpetuate inflammation by secreting chemokines that recruit effector cells and, as professional antigen-presenting cells, by processing and presenting antigens to T cells, thereby bridging innate and adaptive responses (Kmieć et al., 2017).

Within the adaptive immune compartment, imbalances in T and B lymphocytes sustain chronic inflammation and ongoing tissue injury. CD4+ T helper cells exhibit aberrant differentiation; beyond the classic Th2 response, proinflammatory subsets such as Th1, Th9, and Th17 are expanded and hyperactivated. By secreting cytokines including IFN-γ, IL-9, and IL-17, these cells intensify inflammation and disrupt epithelial barrier function (Cao H. et al., 2023; Shohan et al., 2018). Cytotoxic CD8+ T cells are also activated and directly kill epithelial cells through the release of perforin and granzymes (Zhao et al., 2024). Critically, immunoregulatory pathways are compromised. Regulatory T cells (Tregs) display impaired suppressive function and fail to restrain effector T-cell activity (Gu et al., 2024). Concurrently, regulatory B cells (Bregs) are reduced, leading to diminished IL-10 production and further impairing the restoration of immune homeostasis (Wang X. et al., 2016). B-cell heterogeneity is also evident: CD24highCD38high B cells with regulatory potential inversely correlate with disease activity, whereas certain activated B-cell phenotypes are linked to disease exacerbation (Uzzan et al., 2022).

In summary, immune dysfunction in UC comprises exaggerated effector responses coupled with defective regulatory control. This imbalance undermines tolerance to the gut microbiota and, potentially, self-antigens, thereby perpetuating chronic intestinal mucosal inflammation.

### 2.6 An integrated framework for UC pathogenesis

Overall, the pathogenesis of UC reflects a complex interplay among multiple etiologic factors rather than a single determinant. Genetic predisposition confers host susceptibility, whereas environmental exposures act as triggers that disrupt gut homeostasis. These disturbances precipitate microbial dysbiosis, compromise epithelial barrier integrity, and activate aberrant immune responses—processes that interact through continuous bidirectional crosstalk rather than linear pathways. Dysbiosis amplifies barrier dysfunction and immune activation, while inflammation further perturbs microbial composition and impairs epithelial repair, establishing a self-sustaining pathological cycle.

## 3 Concept of probiotics and their mechanisms of action in UC

Probiotics are live microorganisms that, when administered in adequate amounts, confer health benefits to the host. They most commonly include species of *Lactobacillus* and *Bifidobacterium*, as well as certain yeasts (e.g., *Saccharomyces*; Hill et al., 2014; Latif et al., 2023). Because UC is characterized by microbial dysbiosis, epithelial barrier defects, and immune dysregulation, probiotic therapy seeks to restore intestinal homeostasis through coordinated, multi-targeted actions (Guo J. et al., 2024; Hijová, 2025). Proposed mechanisms include competitive exclusion of pathogens and rebalancing of the microbiota (Ronkainen et al., 2025); reinforcement of epithelial barrier integrity (Kim et al., 2025); bidirectional modulation of innate and adaptive immune responses (Si et al., 2025); and attenuation of oxidative stress (Dong et al., 2025). The following sections elaborate on these mechanisms.

### 3.1 Modulation of the gut microbiota

**Figure 2:**
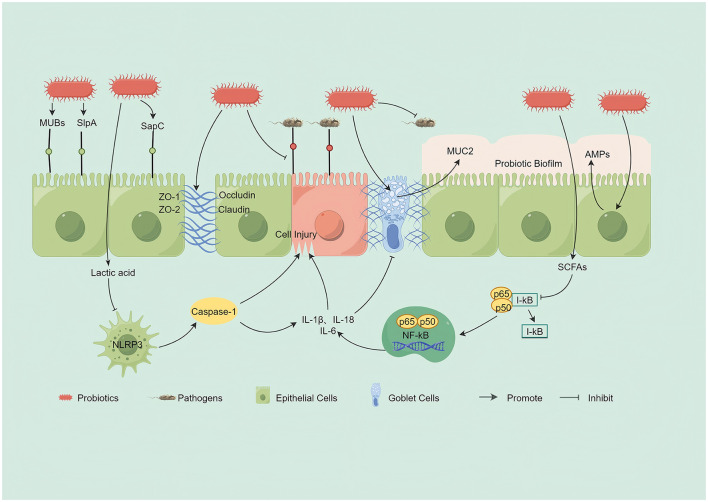
Mechanisms by which probiotics modulate the gut microbiota and strengthen the intestinal barrier: (1) Microbial antagonism: Probiotics compete with pathogens for adhesion sites via adhesins (e.g., MUBs, SlpA, SpaC), secrete antimicrobial peptides (AMPs) and lactic acid, and form protective biofilms. (2) Barrier reinforcement: Probiotics upregulate tight junction proteins (ZO-1, occludin, claudins) and stimulate goblet cells to secrete the mucin MUC2. (3) Inflammation control: Probiotics attenuate NF-κB signaling, reducing the production of IL-6 and TNF-α; inhibit NLRP3 inflammasome activation, thereby decreasing caspase-1 activity and the release of IL-1β and IL-18; and increase the generation of anti-inflammatory short-chain fatty acids (SCFAs). By Figdraw.

Effective intestinal colonization is essential for probiotics to modulate the gut microbiota (Han S. et al., 2021; Figure 2). Colonization occurs primarily through competitive adhesion, whereby probiotic adhesins—including mucus-binding proteins (MUBs), the surface-layer protein SlpA, and the pilus-tip adhesin SpaC—bind with high affinity to epithelial receptors or mucins, thereby spatially excluding pathogenic bacteria such as enteropathogenic *Escherichia coli* and *Salmonella* (Buck et al., 2005; Kankainen et al., 2009; Roos and Jonsson, 2002; Zheng et al., 2023). Beyond physical attachment, adhesion activates host signaling pathways upregulates tight-junction proteins and induce antimicrobial peptide secretion, strengthening the barrier and limiting pathogen invasion (Wang et al., 2025). Following colonization, probiotics further suppress pathogens through ecological and chemical mechanisms, including localized nutrient depletion and secretion of antimicrobial compounds such as bacteriocins (Kommineni et al., 2015). Moreover, specific strains such as *Lactiplantibacillus plantarum* 299v enhance non-heme iron absorption, thereby improving systemic iron status and mitigating UC-associated iron-deficiency anemia (IDA; Ali et al., 2025; Sandberg et al., 2018).

Beyond initial adhesion, some strains form stable biofilm-like assemblies, transitioning from transient colonization to durable niche occupation (van Zyl et al., 2016). This three-dimensional architecture enhances persistence and functions as a local “microfactory,” continuously inducing mucin (e.g., MUC2) production, reinforcing tight junctions, and promoting antimicrobial peptide secretion. Together, these actions fortify the intestinal barrier and consolidate the probiotic ecological advantage (Caballero-Franco et al., 2007; Sicard et al., 2017; van Zyl et al., 2020).

Successful colonization and niche occupation enable probiotics to remodel gut microbial communities. For example, an engineered *Saccharomyces cerevisiae* strain produces lactate that exerts dual actions: it inhibits macrophage NLRP3 inflammasome signaling, thereby attenuating inflammation, and serves as a cross-feeding substrate for select commensals, promoting beneficial taxa (e.g., *Oscillibacter*) while constraining potentially harmful taxa (e.g., *Streptococcus*; Sun et al., 2021). Similarly, *Lactobacillus plantarum* HNU082 enriches *Bifidobacterium pseudolongum* and increases short-chain fatty acids (SCFAs), which strengthens the epithelial barrier and suppresses NF-κB signaling; the resulting milieu limits expansion of pathogens such as *Helicobacter hepaticus* (Wu et al., 2022).

Multi-strain probiotic consortia often provide broader and more robust modulation than single strains. Through complementary functions, they can rapidly suppress pathogens during active inflammation and, during recovery, promote durable colonization and functional restoration of beneficial microbes, thereby more efficiently reestablishing homeostasis across the microbiota–metabolism–immune axis (Xu et al., 2022). By establishing ecological dominance and selectively reshaping microbial composition, probiotics lay the microecological foundation for subsequent barrier repair and immune regulation.

### 3.2 Enhancement of the intestinal barrier

The intestinal mucosal barrier comprises four interdependent layers—physical, chemical, immune, and microbial—and their integrity is essential for intestinal homeostasis and immune balance (An et al., 2022). The physical barrier consists of the epithelial monolayer, intercellular tight junctions, and an overlying mucus layer, which together limit inappropriate translocation of antigens and microorganisms (Allaire et al., 2018; Buckley and Turner, 2018). The chemical barrier is formed primarily by enterocyte-derived molecules, notably antimicrobial peptides (AMPs), that provide non-specific biochemical defense (Mukherjee and Hooper, 2015). The immune barrier is coordinated by gut-associated lymphoid tissue (GALT) and secretory immunoglobulin A (sIgA); sIgA is central to controlling microbial localization and sustaining mucosal tolerance (Donaldson et al., 2018; Macpherson et al., 2018). The microbial barrier—the commensal gut microbiota—confers colonization resistance and shapes the development and functional tuning of the host immune system (Brown et al., 2019; Martens et al., 2018). These layers function in dynamic equilibrium, and disruption of any component can initiate intestinal immune inflammation.

Probiotics repair and fortify these multilayered barriers through multi-target, synergistic mechanisms, and their effects are highly strain-specific (Allam-Ndoul et al., 2025). Different strains preferentially act on distinct barrier layers, creating a complementary network of protection.

Prioritizing the chemical and immune barriers, *Lactobacillus plantarum* HNU082 increases short-chain fatty acid (SCFA) levels, which stimulate goblet cells to secrete MUC2, strengthening the chemical barrier, and reestablishes immune homeostasis by inhibiting NF-κB and bidirectionally modulating cytokine production (Wu et al., 2022; Figure 2).

For direct reinforcement of the physical barrier, a synbiotic comprising *Clostridium butyricum* and chitosan oligosaccharides upregulates tight junction proteins (occludin, claudin-1, ZO-1) and MUC2 while concurrently suppressing TLR4/NF-κB/MAPK signaling, thereby restoring epithelial integrity (Huang et al., 2023).

Acting first on the microbial barrier, *Lactobacillus paracasei* Jlus66 reshapes the gut microbial ecosystem, which in turn inhibits NF-κB/MAPK signaling and downstream pro-inflammatory mediators, ultimately promoting tight junction assembly and function (Yu et al., 2024b).

Collectively, these findings indicate that although probiotics often converge on shared downstream pathways (e.g., anti-inflammatory effects), their primary targets—chemical/immune, physical, or microbial—vary by strain. This multidimensional, networked mode of action provides a rationale for precision microecological interventions tailored to specific barrier defects.

### 3.3 Regulation of immune responses

**Figure 3:**
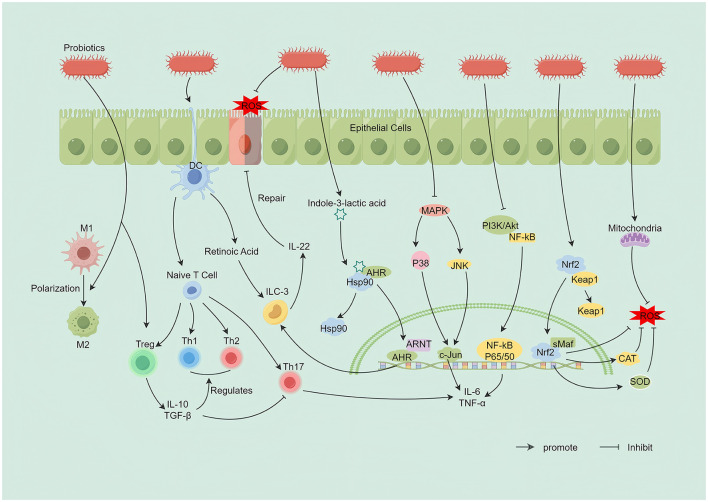
Immunomodulatory and antioxidant mechanisms of probiotics in UC. (1) Probiotics enhance dendritic cell–mediated differentiation of naïve CD4+ T cells, expanding Tregs and restoring immune homeostasis, including a balanced Th1/Th2 response. (2) Probiotics promote macrophage polarization toward the anti-inflammatory M2 phenotype. (3) Probiotic-derived retinoic acid and indole-3-lactic acid, via aryl hydrocarbon receptor (AHR) activation, cooperate to induce group 3 innate lymphoid cells (ILC3s) to produce interleukin-22 (IL-22), thereby supporting epithelial barrier repair. (4) Probiotics inhibit MAPK and NF-κB signaling, reducing proinflammatory cytokines such as interleukin-6 (IL-6) and tumor necrosis factor alpha (TNF-α). (5) Probiotics activate the Nrf2–Keap1 pathway, upregulating antioxidant enzymes including superoxide dismutase (SOD) and catalase (CAT), which limits mitochondrial reactive oxygen species (ROS) and mitigates oxidative injury. By Figdraw.

A central mechanism by which probiotics ameliorate UC is the restoration of intestinal immune homeostasis through multidimensional, systems-level modulation of host immunity. This includes coordinated effects on both innate and adaptive responses and is mediated by engagement of key inflammatory and antioxidant signaling pathways (Figure 3).

First, at the level of innate immunity, probiotics reprogram macrophages and dendritic cells, altering their phenotype and function. Several *Lactobacillus* and *Bifidobacterium* strains drive macrophage polarization from the proinflammatory M1 state toward the anti-inflammatory M2 state, reducing TNF-α and IL-6 while increasing IL-10 (Chen et al., 2025; Su et al., 2024). *Limosilactobacillus reuteri* further promotes CD206+ anti-inflammatory macrophages and robust IL-10 production via activation of the TLR1/2–STAT3 axis (Jia et al., 2022). In addition, ursodeoxycholic acid produced by *Lactobacillus acidophilus* inhibits proinflammatory macrophage polarization by upregulating RapGAP (Deng et al., 2024). Beyond macrophages, *Akkermansia muciniphila* enhances retinoic acid synthesis in dendritic cells, thereby inducing type 3 innate lymphoid cells (ILC3s) to secrete IL-22, a cytokine critical for mucosal repair (Liu H. et al., 2024).

Second, probiotics fine-tune adaptive immune balance, primarily by modulating the abundance and function of T-cell subsets. Certain strains, such as *Lactobacillus paracasei*, suppress differentiation of tissue-injurious Th17 cells while expanding immunosuppressive regulatory T cells (Tregs), thereby increasing mucosal IL-10 and TGF-β and restoring immune tolerance (Chen et al., 2025; Su et al., 2024). Metabolites from *Lactobacillus acidophilus* also promote Treg differentiation (Deng et al., 2024). Other strains, including *Faecalibacterium prausnitzii*, recalibrate Th1/Th2 responses, attenuating excessive Th1 activity (e.g., IFN-γ production) to mitigate immune skewing (Zhou et al., 2025b). In addition, probiotic-derived metabolites such as indole-3-lactic acid activate the aryl hydrocarbon receptor (AhR), upregulating IL-22 and directly promoting epithelial repair (Zhang S. et al., 2025).

The molecular nexus of this multilayered immunomodulation is the coordinated regulation of key intracellular signaling pathways by probiotics. The predominant effect is inhibition of NF-κB, a central inflammatory hub. Diverse strains and their metabolites block NF-κB nuclear translocation, broadly reducing transcription of proinflammatory genes such as TNF-α and IL-6 (Ma Y. et al., 2024; Yu et al., 2024b). For example, vesicles derived from *Lactobacillus rhamnosus* GG inhibit the TLR4–NF-κB axis (Tong et al., 2021). Probiotics also suppress MAPK signaling (e.g., p38, JNK) by limiting phosphorylation, thereby curbing amplification of inflammatory signals (Yu et al., 2024b). Certain strains, including *Lactobacillus acidophilus*, inhibit multiple proinflammatory pathways, such as PI3K–AKT–NF-κB, via their metabolites (Deng et al., 2024). Conversely, probiotics activate cytoprotective programs; notably, they engage the Nrf2 antioxidant pathway to induce endogenous antioxidant enzymes and mitigate the oxidative stress that fuels immune activation (Pan et al., 2025).

In summary, by modulating immune-cell function, rebalancing lymphocyte subsets, and reprogramming core signaling networks, probiotics operate through an integrated cellular-to-molecular network to correct immune dysregulation in UC, offering a multi-target strategy to restore intestinal immune homeostasis.

### 3.4 Alleviation of oxidative stress

In UC, oxidative stress—driven by excessive production of reactive oxygen species (ROS) and impaired antioxidant defenses—propels inflammation and worsens tissue injury (Liu J. et al., 2023). Probiotics counter this pathology through a multitarget, synergistic network that operates at three levels: reducing ROS generation at the source, enhancing ROS scavenging, and reshaping the oxidative microenvironment (Figure 3).

First, probiotics curb ROS production at its origin. For example, *Bifidobacterium longum* lowers basal ROS by improving mitochondrial function in intestinal epithelial cells (Cao F. et al., 2023). Second, they augment ROS clearance by supplying antioxidant molecules—such as glutathione secreted by *Saccharomyces boulardii* (Badr et al., 2021)—and by activating endogenous antioxidant systems. Strains like *Lactobacillus plantarum* increase the activity of superoxide dismutase (SOD) and catalase (CAT) while lowering malondialdehyde (MDA), indicating reduced lipid peroxidation (Din et al., 2020; Liu Q. et al., 2023; Madjirebaye et al., 2024; Pan et al., 2025). Engineered approaches, exemplified by selenium nanodot–modified *Lactobacillus casei* (Se-Lac), further enable targeted delivery of potent antioxidant units to inflamed sites for precise ROS scavenging (Guo P. et al., 2024). Third, probiotics indirectly lessen oxidative burden through systemic regulation: metabolites such as SCFAs suppress excessive inflammation and restore barrier integrity (Khattab et al., 2023), thereby limiting secondary ROS sources, including neutrophil infiltration, and disrupting the vicious cycle of inflammation–oxidative stress.

In sum, by integrating source reduction, direct clearance, and systemic regulation, probiotics offer a comprehensive strategy to mitigate oxidative damage in UC.

### 3.5 Integrated and synergistic network of action

In the intestinal ecosystem, probiotic mechanisms function as an integrated, synergistic network rather than as isolated processes. This network operates as a multidimensional system in which microbiota remodeling initiates the intervention, barrier function provides support, immune regulation drives the response, and antioxidant defenses sustain it (Xu et al., 2025). For example, *Lactococcus lactis* LK mitigates oxidative stress by scavenging reactive oxygen species (ROS) and activating the Nrf2 pathway; remodels the microbiota to suppress pathogens; restores barrier integrity by inhibiting the p53/caspase-3 apoptotic pathway; and reduces lipopolysaccharide (LPS) translocation to downregulate inflammatory mediators. Together, these actions deliver a multitarget synergistic intervention encompassing antioxidant defense, microbiota remodeling, barrier repair, and immune modulation (Sun et al., 2026). The benefits in each component mutually reinforce one another through positive feedback along the microbiota-barrier-immune axis, ultimately driving systemic remodeling of the intestinal microenvironment in UC.

## 4 Application of probiotics in animal models

The therapeutic efficacy of probiotics in UC is well supported by multiple animal models, which enable systematic assessment of barrier restoration, immune modulation, oxidative stress mitigation, and microbiota rebalancing under controlled conditions. Numerous studies show that conventional probiotics act through the synergistic engagement of these pathways. For example, *Lactobacillus plantarum* HNU082 alleviates murine colitis by upregulating the tight-junction protein ZO-1 and the mucin MUC2 while modulating microbial diversity (Wu et al., 2022). *Lactobacillus rhamnosus* GG reduces inflammation by inhibiting the TLR4-NF-κB-NLRP3 axis (Tong et al., 2021). Administration of *Bifidobacterium* species, such as *B. infantis*, increases gut microbial richness and diversity in DSS-induced colitis, with concomitant symptom improvement (Han T. et al., 2021). Yeast probiotics, including *Saccharomyces boulardii*, likewise exhibit barrier-restorative and anti-inflammatory activity across formulations (Jin et al., 2024; Qian et al., 2025).

Summary data in Table 2 highlight shared patterns and genus-specific features of probiotic interventions: (1) Synergy across mechanisms is pervasive. Single strains (e.g., *L. fermentum* 016, *L. plantarum* SC-5) often act on multiple targets simultaneously—enhancing antioxidant defenses (increasing SOD and CAT), repairing the barrier (upregulating ZO-1 and occludin), and modulating immunity (reducing TNF-α and IL-6 while increasing IL-10; Pan et al., 2025; Yu et al., 2024a). (2) Mechanistic strengths vary by genus. Lactobacilli show pronounced direct anti-inflammatory and barrier-restorative effects; bifidobacteria excel at reshaping microbial structure and metabolism (e.g., enriching SCFA producers; Chen et al., 2025; Deng et al., 2024; Fang et al., 2025; Han T. et al., 2021; Lao et al., 2025; Li M. et al., 2025; Pan et al., 2025; Tong et al., 2021; Wu et al., 2023a, 2022; Yang et al., 2025; Yu et al., 2024a); and *Akkermansia muciniphila* engages distinctive immunomodulatory pathways (Liu H. et al., 2024). (3) Engineering approaches (e.g., engineered yeast SyBE 39, bacterial vesicles) can integrate and amplify these effects and add new functions, including targeted delivery and epigenetic modulation (Hao et al., 2021; Nie et al., 2025).

**Table 2:** Application of probiotics in animal studies.

**Probiotic**	**Subjects**	**Duration**	**Regimen (CFU)**	**Core mechanisms**	**Key findings**	**Ref**.
Lactobacillus						
*Lactobacillus plantarum* HNU082	C57BL/6 mice	7-15d	1 × 10^9^CFU/mL	Barrier repair, microbiota modulation	↑ Body weight, colon length, ZO-1, MUC-2, microbiota diversity, SCFA-producing genera; ↓ DAI, immune organ index, cytokines, tissue injury	(Wu et al. 2022)
*Lacticaseibacillus paracasei* L21	C57BL/6J mice	7d	5 × 10^9^ CFU/mL	Barrier repair, microbiota modulation, immunity (AhR/IL-22)	↑ Body weight, colon length, ZO-1, MUC2, SCFAs, *Akkermansia*, microbiota diversity; ↓ DAI, spleen index, MPO, cytokines, serum LPS	(Chen et al. 2025)
*Lactobacillus acidophilus*	C57BL/6J male	7d	2 × 10^9^CFU/mL	Immunomodulation (anti-inflammatory)	↑ IL-10, TGF-β; ↓ TNF-α, IL-1β, IL-6	(Deng et al. 2024)
*Lactobacillus rhamnosus* GG	C57BL/6J male	2w	1.2 mg/kg	Anti-inflammation (TLR4-NF-κB-NLRP3)	↑ Colon length; ↓ TNF-α, IL-1β, IL-6, IL-2, *Proteobacteria*	(Tong et al. 2021)
*Lactobacillus fermentum* 016	C57BL/6 mice	28d	1 × 10^9^ CFU/day	Antioxidant (Nrf2), barrier repair, microbiota modulation	↑ Body weight, colon length, barrier proteins, anti-inflammatory cytokines, beneficial bacteria, tryptophan metabolites, T-SOD, GSH-Px, CAT; ↓ DAI, spleen index, tissue injury, cytokines, MDA, MPO	(Pan et al. 2025)
*Lactobacillus plantarum* SC-5	C57BL/6 mice	7 d	1.0 × 10^10^ CFU/kg/day	Antioxidant, barrier repair, microbiota modulation	↑ Body weight, colon length, tight junction proteins, T-SOD, CAT, GSH-PX, beneficial bacteria; ↓ DAI, cytokines, MPO, MDA	(Yu et al. 2024a)
*Lacticaseibacillus rhamnosus* G7	C57BL/6J mice	15d	1 × 10^9^CFU/mL	Anti-inflammation, Microbiota modulation	↑ IL-10, *Faecalibaculum*; ↓ DAI, IL-1β, IL-6, TNF-α, *Bacteroides, Escherichia-Shigella*	(Lao et al. 2025)
*Lactiplantibacillus plantarum* HYY-S10	C57BL/6J mice	7d	1 × 10^8^CFU/mL	Antioxidant, Anti-inflammatory	↑ IL-10, SCFA producers, GPx; ↓ DAI, IFN-γ, IL-1β, IL-6, LPS, MPO, NO	Li M. et al. (2025)
*Lactobacillus acidophilus (LA)*	BALB/c mice	5d	1 × 10^8^ CFU/day	Barrier repair, immunomodulation, microbiota modulation	↑ Body weight, colon length, ZO-1, Occludin, IL-10, TGF-β, beneficial bacteria; ↓ DAI, cytokines	(Fang et al. 2025)
Bifidobacterium						
*Bifidobacterium infantis*	C57BL/6 mice	14d	1.5 × 10^9^CFU/mice QDCFU/mL	Microbiota modulation, anti-inflammatory	↑ Microbial richness; ↓ UC symptoms, DSB levels	Han T. et al. (2021)
*Bifidobacterium animalis* A6	BALB/c mice	14d	1.5 × 10^9^ CFU/mouse/day	Barrier repair, Metabolism (butyrate)	↑ FFAR2/3 expression, butyrate metabolism; ↓ UC symptoms, IL-13, claudin-2	(Wu et al. 2023a)
*Bifidobacterium infantis* EVC001	C57BL/6J male	3 w	1 × 10^10^ CFU/day	Barrier repair, Immunomodulation, Microbiota modulation	↑ Body weight, colon length, tight junction proteins, MUC2, *Akkermansia, Bifidobacterium*; ↓ DAI, spleen index, tissue injury, serum LBP, cytokines	(Yang et al. 2025)
Yeast						
Heat-killed *Saccharomyces boulardii*	C57BL/6J mice	8d	1 × 10^7^ CFU/0.2 mL	Barrier repair, Anti-inflammatory	↑ Gut barrier; ↓ UC symptoms, TNF-α, IL-1β	(Jin et al. 2024)
*S. boulardii* (SB@TA-Mg^2+^@CPP)	C57BL/6J mice	14d	1.5 × 10^8^ CFU/mouse/day	Barrier repair, Anti-inflammatory	↑ ZO-1, Occludin, MUC2, serum Mg^2+^; ↓ DAI, TNF-α, IL-6, IL-1β	(Qian et al. 2025)
*Saccharomyces boulardii*	C57BL/6J mice	3 w	1 × 10^6^ CFU/kg/day	Anti-inflammatory	↑ Body weight, colon length; ↓ Colitis, TNF-α, IL-6, IL-1β	(Gao et al. 2023)
*S. boulardii* FN (Fibronectin-targeted)	C57BL/6J mice	Single dose (day 5)	1.5 × 10^9^ CFU/dose	Targeted delivery, Anti-inflammatory	↑ colon length, IL-10; ↓ Histological score, TNF-α	(Heavey et al. 2024)
Other genera						
*Akkermansia muciniphila* (live)	C57BL/6J mice	14 d	200 μL suspension	Immunomodulation (DCs/RA)	↑ IL-22, IL-10, CD103^+^CD11b^−^ DCs, RALDH2, RA; ↓ DAI, colon shortening, tissue injury, permeability, TNF-α, IL-6	Liu H. et al. (2024)
*Pediococcus acidilactici*	C57BL/6 mice	21 d	1 × 10^9^ CFU/0.2 mL/day	Antioxidant, anti-inflammatory	↑ Body weight, colon length, thymus index, SOD, GSH-Px, T-AOC; ↓ DAI, spleen index, tissue injury, cytokines, MDA, MPO, NOS	(Wang et al. 2024)
*Bacillus natto* JLCC513	C57BL/6 mice	14 d	1 × 10^10^ CFU/ml	Anti-inflammatory (LPS/TLR4/NF-κB), Barrier repair, Microbiota modulation	↑ Body weight, colon length, tight junction proteins, beneficial bacteria; ↓ DAI, tissue injury, cytokines (IL-1, IL-6, TNF-α), MDA	Ma M. et al. (2024)
Multi-strain						
VSL#3 (*L. casei, L. plantarum, L. acidophilus, L. bulgaricus, B. longum, B. breve, B. infantis, S. thermophiles*)	Mice	12d	1.5 × 10^9^ CFU/mouse/day	Anti-inflammatory (NF-κB)	↓ Tumor burden, TNF-α, IL-6, NF-κB & TCF-4 activity	Li W. et al. (2022)
*Tetragenococcus halophilus, Eubacterium rectale*	C57BL/6 mice	10 d	T. halophilus: 3.8 × 10^8^ CFU/day; E. rectale: 1.0 × 10^8^ CFU/day	Immunomodulation, Microbiota modulation	↑ Body weight, colon length, FoxP3, IL-10, beneficial bacteria, *Actinobacteria, Verrucomicrobia*; ↓ Histological inflammation, TNF-α, DC markers, CD8^+^NK1.1^+^ cells, neutrophils, *Proteobacteria*, pathobionts	(Ryu et al. 2024)
Engineered bacteria/derivatives						
Engineered yeast SyBE 39	C57BL/6 male	7 d	/	Immunomodulation (macrophages), epigenetics	↑ Colon length, ZO-1, MUC2, M2 macrophages, H3K9ac, H3K18la, microbiota diversity, beneficial bacteria; ↓ Disease activity, spleen index, histology, cytokines, M1 macrophages, NLRP3, pyroptosis (GSDMD), harmful bacteria	(Sun et al. 2021)
*L. plantarum* Q7-derived vesicles	C57BL/6J male	18 d	10, 20 μg	Anti-inflammatory (TLR4/NF-κB), microbiota modulation	↑ Colon length, diversity, beneficial bacteria; ↓ Disease activity, spleen index, tissue injury, cytokines, TLR4/MyD88/NF-κB pathway, harmful bacteria	(Hao et al. 2021)
*B. longum* NSP001-derived EVs (NEVs)	C57BL/6 mice	14 d	150 μg/mL/day	Immunomodulation (Th17/Treg), barrier repair, microbiota-dependent/independent	↑ Body weight, colon length, goblet cells, MUC2, tight junction proteins, IL-10, TGF-β, Tregs, SCFAs, microbiota structure; ↓ DAI, spleen index, tissue injury, cytokines, MPO, macrophage infiltration, Th17, p-STAT3	(Nie et al. 2025)

The dosing regimen (CFU: colony-forming unit) is listed as reported in the original studies, including daily CFU per animal or concentration of bacterial suspension. Specific administration volumes can be found in the cited references.

Current research has moved beyond demonstrating the efficacy of single strains and now focuses on designing multimodal, precision interventions, marking a shift from discovery to design. These strategies include: (1) Engineered probiotic strains: Genetic modification endows probiotics with new therapeutic functions. For example, (Zhou et al. 2022) engineered *Escherichia coli* Nissle 1917 to stably express catalase and SOD, enabling active scavenging of intestinal reactive oxygen species, thereby more effectively repairing the epithelial barrier and modulating the microbiota. Clinical translation of such strains is constrained by stringent regulations in some jurisdictions, including the European Union (von Wright, 2005), yet they remain invaluable research tools for elucidating mechanisms and hold promise for future use in tightly controlled medical settings. (2) Smart delivery systems: Novel carriers enable targeted delivery and controlled release of probiotics or drugs. Li L. et al. (2025) used yeast cell wall microparticles to coencapsulate *Bacillus subtilis* and rhein—an anthraquinone from rhubarb with anti-inflammatory activity (Fu et al., 2024)—delivering both agents to the colonic submucosa and promoting tissue repair by activating local neural pathways. (3) Neuroimmune co-regulation: this approach simultaneously addresses intestinal inflammation and associated nervous system complications. Zhang X. et al. (2025) designed an 18β-glycyrrhetinic acid prodrug nanomicelle that, when combined with *Lactobacillus rhamnosus* GG, repaired the intestinal barrier and inhibited microglia-mediated neuroinflammation, thereby alleviating UC and concomitant depressive-like behaviors. (4) Synergistic combinations of strains and bioactive components: this strategy leverages supra-additive (1 + 1 > 2) effects between probiotics and functional compounds. (Sharma et al. 2025) showed that probiotic fermentation of Indian gooseberry juice significantly increased the bioavailability of flavonoids through biotransformation. The resulting fermentation-enriched metabolites act on multiple nodes of the gut–immune axis, synergistically suppressing pro-inflammatory factors (e.g., TNF-α) and enhancing antioxidant enzyme activity (e.g., SOD), thereby more effectively mitigating colitis.

In summary, probiotic research in UC animal models has progressed from basic efficacy testing to precision interventions that integrate bioengineering, targeted delivery, and systems-biology approaches. This evolution provides a strong foundation for developing next-generation UC therapies. However, methodological challenges in inducing chronic colitis and welfare-related limits on study duration have kept long-term colonization studies (>4 weeks) relatively rare (Table 2). Consequently, most interpretations of probiotic efficacy and safety from animal models pertain to short-term interventions. Long-term colonization dynamics and durable therapeutic effects should be investigated using refined models and extended follow-up.

## 5 Clinical application of probiotics

The clinical utility of probiotics in UC is primarily as an adjunct to standard therapy, with evidence of synergistic benefit in selected contexts. High-quality randomized controlled trials indicate that adding specific formulations—such as *Escherichia coli* Nissle 1917—to mesalazine (5-aminosalicylic acid [5-ASA]; a standard anti-inflammatory agent for UC; Brogden and Sorkin, 1989) can further improve clinical remission and mucosal healing, underscoring their adjuvant value (Agraib et al., 2022; Park et al., 2022). However, efficacy varies markedly by strain, formulation, dosing regimen, host characteristics, and disease activity, and not all probiotic products are effective. In addition, integrative approaches—such as combining probiotics with digital health–supported management—may enhance broader outcomes, including nutritional status, systemic inflammation, and quality of life (Ou et al., 2021; Rayyan et al., 2023).

Based on the clinical studies summarized in Table 3, several conclusions emerge. First, as adjuncts to standard care, selected strains or formulations—such as *Escherichia coli* Nissle 1917, VSL#3, and certain multi-strain probiotics—provide additive or synergistic benefits, improving key outcomes including clinical response, endoscopic remission, and health-related quality of life (HRQoL; Agraib et al., 2022; Park et al., 2022; Rayyan et al., 2023; Tursi et al., 2010). Second, benefits extend beyond intestinal symptoms to broader HRQoL domains, including emotional and social functioning (Rayyan et al., 2023). Third, synbiotics (probiotic–prebiotic combinations) may support mucosal microrepair with long-term use (Ishikawa et al., 2011). Nonetheless, efficacy is varies across products and patient subgroups. For example, *Trichuris suis* ova therapy was ineffective overall but showed benefit in specific subgroups (e.g., patients not receiving corticosteroids; Prosberg et al., 2024). These findings argue against a one-size-fits-all approach and underscore the need for personalized treatment strategies.

**Table 3:** Clinical Applications of Probiotics.

**Probiotic (regimen)**	**Control**	**Participants (T/C)**	**Length and route of probiotic application**	**Study design**	**Primary outcome**	**Population**	**Ref**.
Synbiotic *(Bifidobacterium longum* 2 × 10^11^ CFU + 6 g prebiotic synergy 1, BID*)*	Placebo (potato starch capsule + 6 g maltodextrose)	8/8 (16 completed)	4 weeks orally	Randomized, double-blind, placebo-controlled pilot trial	Positive: Significant reduction in mucosal inflammatory markers and improved endoscopic/histologic scores.	UK	(Furrie et al. 2005)
*Escherichia coli* Nissle 1917 (EcN) (daily)	Placebo	58/60	8 weeks orally	Multicenter, double-blind, randomized trial	Positive (adjunct): Significantly prevented IBDQ deterioration; improved clinical response and endoscopic remission rates.	South Korea	(Park et al. 2022)
Multi-strain capsule (9 *Lactobacillus* spp., 5 *Bifidobacterium* spp.) (3 × 10^10^ CFU, TID)	Placebo (polysaccharide)	12/12	2 years orally	Randomized, double-blind, controlled trial	Positive: Significantly induced clinical remission; improved partial Mayo score and inflammatory markers.	Jordan	(Agraib et al. 2022)
*Bifidobacterium* triple viable powder + WeChat-based management	Routine follow-up	75/75	12 weeks orally	Randomized controlled trial	Positive: Significantly improved nutritional status, reduced inflammatory cytokines, enhanced quality of life.	China	(Ou et al. 2021)
Multi-strain capsule (9 *Lactobacillus* spp., 5 *Bifidobacterium* spp.) (3 × 10^10^ CFU, TID)	Placebo	12/12	6 weeks orally	Randomized, double-blind, controlled trial	Positive: Significantly improved quality of life (all domains and total SIBDQ score).	Jordan	(Rayyan et al. 2023)
*Bifidobacterium infantis* 35624 (1 × 10^10^ CFU/day, in sachet)	Placebo (maltodextrin)	13/9	6 weeks orally	Randomized, double-blind, placebo-controlled trial	Positive: Significantly reduced plasma CRP (*p* = 0.0327) vs. placebo; trend toward reduced IL-6 (*p* = 0.057)	Ireland	(Groeger et al. 2013)
VSL#3 (3.6 × 10^12^ CFU/day, BID)	Placebo	71/73	8 weeks orally	Randomized, double-blind, controlled trial	Positive (adjunct): Significantly increased clinical response rate (UCDAI reduction ≥50%).	Italy	(Tursi et al. 2010)
*B. breve* Yakult + GOS (Synbiotic) (1g probiotic powder TID + 5.5g GOS QD)	No intervention	20/21	12 months orally	Randomized controlled trial	Positive: Long-term treatment significantly improved colonoscopy scores and inflammatory markers.	Japan	(Ishikawa et al. 2011)
*Trichuris suis* ova (TSO) (7500 ova, biweekly)	Placebo	60/59	24 weeks orally	Randomized, double-blind, controlled trial (phase 2b)	Negative: No significant difference in clinical remission rate vs. placebo.	Denmark	(Prosberg et al. 2024)
*Ligilactobacillus salivarius, Lactobacillus acidophilus, Bifidobacterium bifidum* BGN4(3 × 10^9^ CFU/day)+ 1,200 mg/day mesalazine	1,200 mg/day mesalazine alone	30/30	2 years orally	Controlled trial	Positive: improved Mayo score, reduced stool frequency, enhanced mucosal healing; benefits sustained after 2 years	Italy	(Palumbo et al. 2016)

The observed heterogeneity in efficacy reflects complex interactions across multiple levels: host factors (genetic background, baseline microbiota, immune status); intervention factors (strain specificity, formulation and viability, dose, duration); and trial design factors (endpoint selection, follow-up length). Consequently, although adjuvant benefits have been demonstrated for formulations such as VSL#3 (Tursi et al., 2010), and synbiotics can modulate the microbiota and improve mucosal status (Ishikawa et al., 2011), the durability of these effects and the optimal implementation strategy—including patient selection, timing, and combinations—remain uncertain. Rigorous, biomarker-stratified studies are needed to resolve these questions.

In summary, the role of probiotics in UC management is shifting from “alternative therapy” to adjuncts that augment standard treatment. From the perspective of clinical translation, existing evidence provides preliminary support for the feasibility of this shift—current human trials typically employ administration periods exceeding 4 weeks (Table 3), establishing a foundation for evaluating long-term efficacy and safety. However, for probiotic therapies to be truly integrated into clinical practice, three practical challenges must still be addressed: verification of long-term safety, optimization of cost-effectiveness, and assurance of patient compliance.

## 6 Emerging probiotic strategies

To address limitations of conventional live probiotics—such as low survival through the gastrointestinal tract, variable colonization, and inconsistent efficacy—intervention strategies are shifting toward non-viable derivatives, multimodal combinations, precision delivery platforms, and reconstitution of the gut ecosystem. This chapter highlights four cutting-edge directions: postbiotics, probiotic–traditional Chinese medicine (TCM) combinations, targeted delivery systems, and fecal microbiota transplantation (FMT).

### 6.1 Postbiotics

Postbiotics are bioactive metabolites and cellular components produced by probiotics during fermentation or after cell lysis, representing a microbiome-based intervention that extends beyond live bacteria. Compared with traditional live probiotics, postbiotics offer advantages—defined composition, improved stability, potentially greater safety, and more tractable mechanisms of action—yet they also have inherent limitations (Pattapulavar et al., 2025). These core characteristics are systematically compared in Table 4.

**Table 4:** Comparison of characteristics between postbiotics and traditional viable probiotics.

**Feature**	**Traditional viable probiotics**	**Postbiotics**	**Ref**.
Composition	Live microbial cells; complex and subject to batch-to-batch variations	Defined metabolites or cell components (e.g., SCFAs, enzymes, exopolysaccharides); quality-controllable	(Pattapulavar et al. 2025)
Stability	Sensitive to temperature, humidity, gastric acid, and bile salts; low survival rate	Acid-resistant, heat-stable; easy storage and transport; extended shelf life	(Amobonye et al. 2025)
Mechanism of action	Require colonization to exert effects via metabolites or host interactions	Directly act on intestinal epithelium and immune cells	(Pattapulavar et al. 2025)
Colonization capacity	Certain strains can establish short- or long-term colonization, forming ecological niches	No colonization capacity; require continuous supplementation to maintain effects	(Pattapulavar et al. 2025)
Safety	Risk of bacteremia (particularly in immunocompromised patients)	No live cell-related risks; suitable for immunocompromised individuals	(Meini et al. 2015); (Vahabnezhad et al. 2013)
Therapeutic consistency	Subject to host baseline microbiota, gastric acid degradation, and other factors; significant inter-individual variability	Fixed composition; predictable effects; high batch-to-batch consistency	(Chen et al. 2025); (Estevinho et al. 2024)

As summarized in Table 4, postbiotics preserve key probiotic activities in a cell-free form while avoiding several limitations of live preparations. However, they lack the capacity to colonize the gut and provide sustained stimulatory effects characteristic of viable strains. Accordingly, their mode of action is better described as exogenous supplementation rather than endogenous reconstitution, implying that ongoing administration may be required to maintain efficacy.

In terms of specific active ingredients, SCFAs are the most extensively studied postbiotics. Butyrate and propionate fuel colonic epithelial cells and strengthen barrier function (Liu C. et al., 2024; Zhang J. et al., 2025). They also enhance mucin secretion and help restore immune homeostasis by inhibiting NF-κB and activating signaling pathways such as HIF-1α and AhR (Chen et al., 2025). Other active components also show benefit. The cell-free supernatant of *Bacillus amyloliquefaciens* C-1 suppresses pro-inflammatory mediators, including IL-1β and TNF-α, through antioxidant and antibacterial activities (Hou et al., 2025). Heat-inactivated preparations from *Lacticaseibacillus rhamnosus* 1.0320 reduce inflammation and oxidative stress by inhibiting the TLR4/MAPK/NF-κB pathway (Zhang J. et al., 2025). Notably, several studies report that specific postbiotics can match or even surpass their parent live strains in improving disease activity indices, repairing the intestinal barrier, and mitigating oxidative stress (Rezaie et al., 2024a,b, 2025; Ye et al., 2024). These findings indicate that postbiotics are not simply a downgraded surrogate for live probiotics but rather concentrate and potentiate key bioactive components. For patients unable to tolerate live preparations—such as immunocompromised or critically ill individuals—or those requiring long-term, stable therapy, postbiotics offer a safe, controllable alternative.

### 6.2 Probiotic–traditional Chinese medicine combinations

The multi-component, multi-target properties of TCM can be leveraged with the microecological modulation of probiotics to create a synergistic, comprehensive therapeutic strategy. Mechanistically, combination therapies exert multi-pathway effects. For example, *Lactobacillus* combined with the TCM compound HKL (a multi-herb anti-inflammatory formulation; Han Z. et al., 2021) modulates the gut microbiota and inhibits the TLR9 pathway (Aximujiang et al., 2022), while *Lactobacillus brevis* with a saponin extract synergistically suppresses NF-κB signaling and the release of pro-inflammatory cytokines (Kim et al., 2015). Probiotic fermentation also enhances the bioavailability and immunomodulatory activity of bioactive constituents from TCM herbs, including ginseng and *Salvia miltiorrhiza* (Kim et al., 2017; Park et al., 2016; Qu et al., 2021; Su et al., 2021). At the systems level, combinations of probiotics with herbs such as *Codonopsis pilosula* and red ginseng promote intestinal homeostasis by facilitating colonization of beneficial taxa, increasing short-chain fatty acid production, and rebalancing the Th17/Treg axis (Guo et al., 2015; Jing et al., 2018; Wang et al., 2020; Yun et al., 2020). Clinically, a meta-analysis of 14 randomized controlled trials (*n* = 1,154) found that probiotic–TCM combinations were superior to 5-ASA monotherapy, probiotic monotherapy, or TCM monotherapy in inducing clinical remission, reducing relapse rates, and lowering adverse events (Hu et al., 2022). In summary, probiotic–TCM combinations capitalize on multi-target, systems-level regulation and represent a promising clinical strategy. Future research should elucidate principles of strain–herb compatibility to enable precise, standardized application.

### 6.3 Targeted delivery systems

To address the limitations of oral administration, novel delivery platforms have been developed around three objectives: preserving probiotic viability and promoting colonization, achieving precise targeting of inflamed sites, and enabling synergistic, multipathway therapy.

First, encapsulation is key to enhancing survival and colonization. Metal–phenolic network (MPN) coatings protect lactobacilli from gastric acidity and substantially increase mucoadhesion in the intestinal mucus layer (Gao L. et al., 2025). Likewise, double-layer alginate microcapsules encapsulating *Lactobacillus plantarum* with an inner core of prebiotic resistant starch enhance bacterial proliferation and metabolic activity in the colon (Liu M. et al., 2024). Emerging microfluidic platforms enable co-encapsulation of probiotics with prebiotics (e.g., alginate–inulin hydrogels) within microparticles, conferring resistance to gastrointestinal stress, promoting colonic retention, and supporting *in situ* short-chain fatty acid fermentation, thereby augmenting anti-inflammatory effects (Yin et al., 2024). Collectively, these encapsulation strategies improve the oral bioavailability of probiotics by providing physical protection and nutritional synergy.

Second, to improve targeting specificity to inflammatory foci, stimuli-responsive systems can sense features of the inflammatory microenvironment and trigger on-demand release. For example, functionalizing *Lactobacillus rhamnosus* GG with ROS-responsive nanomicelles enables targeted delivery of 18β-glycyrrhetinic acid at inflamed sites, thereby scavenging excess ROS and repairing the epithelial barrier (Zhang X. et al., 2025). Similarly, metabolic engineering of *Escherichia coli* Nissle 1917 (EcN-TRP), combined with double-layer microcapsule delivery, achieves colon targeting and elevates local levels of immunomodulatory indole metabolites (e.g., indole-3-propionic acid) (Li et al., 2024). A growing number of studies also endow probiotics with active homing via surface functionalization. For instance, hyaluronic acid (HA)-modified “nano-armor” protects probiotics during gastric transit and enables precise targeting through HA-CD44 interactions, as CD44 is highly expressed at inflamed sites; subsequent microenvironment-triggered degradation of the coating releases the probiotics at the target site, enabling timely therapeutic action (Zhu L. et al., 2024).

Recent work is advancing multifunctional composite platforms that use probiotics as living carriers to co-deliver multiple therapeutics, enabling synergistic, multi-pathway interventions at sites of inflammation. Representative nanoengineering strategies include pH-responsive alginate coatings that co-encapsulate *Escherichia coli Nissle* 1917 (EcN) with 5-aminosalicylic acid, enabling simultaneous intestinal release of live bacteria and drug to modulate the microbiota, suppress pro-inflammatory cytokines, and repair the intestinal barrier (Peng et al., 2023); bilirubin-loaded EcN conjugated with hyaluronic acid (EcN–BR/HA), which integrates ROS-scavenging, immunomodulatory, and microbiota-balancing activities (Peng et al., 2025); and bioorthogonal conjugation of immunomodulators to the EcN surface, where dissolution of an enteric coating in the intestine exposes the conjugates to drive macrophage polarization from pro-inflammatory M1 to anti-inflammatory M2, complementing the probiotic's intrinsic effects (Peng et al., 2024). Beyond engineering live probiotics, their derivatives—such as exosomes and membrane vesicles—provide platforms for delivering nucleic acid therapeutics and nanozymes. *Lactobacillus*-derived exosomes have been used as nanocarriers for targeted delivery of TNF-α siRNA to intestinal inflammatory cells, effectively silencing this key pro-inflammatory cytokine (Cui et al., 2025). In another study, membrane vesicles from *Escherichia coli* Nissle 1917 (EcN) were co-loaded with manganese dioxide (MnO_2_) nanozymes and the phosphodiesterase-4 inhibitor roflumilast (Song et al., 2024; Wedzicha et al., 2016); the resulting construct increased intracellular cyclic adenosine monophosphate (cAMP) in macrophages, thereby suppressing TNF-α production and favorably remodeling the gut microbiotat.

### 6.4 Fecal microbiota transplantation

Fecal microbiota transplantation (FMT) has emerged as a broad-spectrum microecological intervention for UC, with notable therapeutic advances in recent years. Unlike the targeted supplementation approach of probiotics, FMT aims to reconstitute a dysbiotic gut ecosystem by transferring a complete microbial community from healthy donors (Ren et al., 2025; Singhal et al., 2025). Mechanistic studies link efficacy to engraftment of beneficial taxa (e.g., *Eubacterium hallii, Roseburia* spp.) and restoration of short-chain fatty acid levels (Xu et al., 2021). Multiple clinical trials show that FMT is superior to placebo for inducing clinical remission in active UC (Doukas et al., 2025; Paramsothy et al., 2019), although outcomes are influenced by donor selection, delivery route, and the patient's baseline microbiota (Caenepeel et al., 2025; Chen et al., 2023; Pinto et al., 2025). Persistent challenges include donor standardization, long-term safety, and reproducibility of efficacy. Nevertheless, FMT's holistic reconstruction paradigm complements the targeted probiotic strategy, together expanding the therapeutic armamentarium for UC.

## 7 Summary and outlook

Disruption of gut microbial homeostasis is a major driver of UC onset and progression. Microbiome-directed probiotic interventions have shown promise in preclinical and clinical studies, ameliorating UC through multiple mechanisms, including anti-inflammatory activity, epithelial barrier restoration, and remodeling of the intestinal microbiota. However, clinical translation remains limited by key challenges: low colonization efficiency of conventional oral formulations in the complex gastrointestinal milieu; substantial interindividual variability in therapeutic response; and the lack of adaptive, stage-specific intervention strategies.

Future research should progress along three fronts. First, innovate delivery by engineering probiotics and developing smart, targeted platforms to enhance the survival, colonization, and retention of live bacteria at inflamed colonic sites. Second, broaden therapeutic modalities by creating non-viable biotherapeutics—such as postbiotics—with well-defined, stable compositions to overcome the limitations of live microbes and achieve more consistent efficacy. Third, design precision combination strategies, including integration of probiotics with TCM to leverage its multitarget regulatory properties, and personalize treatment plans based on host–microbiome profiles. Finally, advance fecal microbiota transplantation (FMT) through donor-recipient matching models, defined microbial consortia, and standardized protocols to improve efficacy and reproducibility.

Collectively, these advances will shift probiotic therapy from a broad-spectrum supplement to a precision modulator, delivering efficient, stable, and personalized next-generation microecological treatments for the clinical management of UC.
